# Food for Thought: Exploring Dental Students' Perceptions of Delivering Dietary Advice to Children and Families

**DOI:** 10.1111/eje.13118

**Published:** 2025-05-13

**Authors:** Helen Rogers

**Affiliations:** ^1^ School of Dental Sciences, Faculty of Medical Sciences Newcastle University Newcastle Upon Tyne UK

**Keywords:** dental education, dietary advice, prevention, qualitative

## Abstract

**Background:**

Delivery of dietary advice by dental professionals is an important part of national guidelines, to prevent dental disease and improve general health in children and young people. Anecdotally, dental students feel uneasy delivering dietary advice to children and their parents/carers, which may affect their ability to provide effective advice confidently. This study aimed to explore undergraduate dental students' perceptions of delivering dietary advice to children and parents/carers.

**Method:**

Semi‐structured qualitative interviews were held with undergraduate dental students who had experience of providing dietary advice for children and parents. Interviews were steered using a topic guide, and were recorded and transcribed verbatim. Recruitment continued until data saturation was considered to have been achieved. Two researchers undertook thematic analysis on the transcriptions independently, prior to agreeing upon the key themes and subthemes.

**Results:**

Interviews were carried out with 11 students with varied levels of experience in providing dietary advice. Managing the child–parent dyad was a key theme, with students finding it challenging to tailor age‐appropriate information to engage both parties, particularly when the child's behaviour became difficult. Students found it challenging to deliver dietary advice to children and parents from diverse cultural backgrounds and were aware that families may struggle to implement the advice that they were delivering due to financial difficulties and complex childcare arrangements. Students felt their learning experience in this area was adversely affected by the pandemic.

**Conclusion:**

This is the first study to explore student perceptions of delivering dietary advice, providing rich data to inform future teaching developments and research.

## 1 Introduction

Untreated dental caries (tooth decay) is the most common disease to affect children globally [1], with significant impacts on their quality of life and wellbeing [2]. Removal of carious teeth remains the most common reason for a child in England to be admitted to hospital [3]. Caries occurs due to acid demineralisation of tooth tissue, produced as a by‐product of bacterial metabolism of sugars residing in dental plaque [4]. As such, the reduction of the frequency of children's consumption of free sugars is a priority in both preventing the disease from occurring and slowing its progression [5]. Provision of dietary advice to reduce sugar intake is hence a key component of national Delivering Better Oral Health guidance [6].

Newcastle University runs an undergraduate bachelor's in Dental Surgery (BDS) degree. This course involves a mandatory assessment of clinical competency in dietary advice delivery. This is also a requirement of the curriculum framework prescribed by the UK dental regulatory body, the General Dental Council, which ensures that a student is working at the level expected of a safe practitioner upon graduation [7].

Within the context of Newcastle University, students deliver dietary advice to children and families in a bay on an open plan clinic. Dietary advice is typically tailored to patients following receipt of a three‐day paper diet diary which is completed by families at home, which is subsequently analysed for the frequency of sugar consumption. This is followed by administering paper‐based resources, including a child‐friendly ‘Garfield’ leaflet, with space for take home messages to be added by the student.

Anecdotally, the delivery of dietary advice can be challenging. The literature suggests that dietary advice is rarely provided by qualified general dental practitioners (despite national guidance) and is limited when it is provided [8]. This is broadly attributed to a lack of financial remuneration, time constraints and a lack of education and training on the topic for dental practitioners [8]. No existing research has been undertaken to explore dental students' perceptions of delivering diet advice in paediatric dental clinics, though this has been recommended within the literature [8]. Greater understanding of student perceptions may facilitate improvements to education and training on this topic, which in turn could increase the number of dental practitioners continuing to provide dietary advice post‐qualification.

This study aimed to qualitatively explore the perceptions of undergraduate dental students surrounding dietary advice delivery in paediatric dental clinics. The specific objectives were to:

- explore students' perceptions of delivering dietary advice to children and parents/carers
- seek students' insights into their experiences of learning to deliver dietary advice
- seek students' suggestions on how they could be further supported to deliver advice confidently and effectively

This manuscript has been written in accordance with the consolidated criteria for reporting qualitative research checklist (COREQ) [9].

## 2 Method

Ethical approval for this study was granted by the Faculty of Medical Sciences Ethics Committee at Newcastle University (reference: 2248/16176). A qualitative methodology was chosen for this study to enable the generation of rich data using one‐to‐one, online interviews, with inductive thematic analysis.

### 2.1 Sample

A purposive sample was sought to enable exploration of a variety of experiences from students across different year groups and courses. The examinations team within the Dental School was consulted to ensure the study did not interfere with the revision period for examinations in these year groups.

#### 2.1.1 Inclusion Criteria

- Stage 4 or Stage 5 BDS student.
- Delivered dietary advice to children and parents on at least one occasion.

#### 2.1.2 Exclusion Criteria

- No previous experience of delivering dietary advice to children and parents.

### 2.2 Recruitment

Open invitations were emailed to two purposively sampled clinical groups where all students were known to meet the aforementioned criteria. Each clinical group comprised between 8 and 10 students. Students that responded to the invitation to take part were sent a participant information sheet and a consent form via email, which they were asked to return completed electronically. Interviews were arranged at a time convenient for the students.

Recruitment continued until data saturation had been reached, whereby no new ideas or concepts were being identified during analysis [10].

### 2.3 Interviews

Interviews were conducted on Microsoft Teams (Microsoft Corporation, Washington, United States) by one interviewer. This was to maximise flexibility for participants, to allow them to undertake their interview around their clinical timetables. Consent was confirmed immediately prior to the interview. Participants were advised that they could stop the interview and withdraw from the study at any time.

The interviews were steered using a topic guide (see Supporting Information) that was developed iteratively following each subsequent interview to prompt deeper exploration of ideas arising from previous interviews. Interviews were anticipated to last between 20 and 30 minutes.

All interviews were recorded using the online platform and a digital voice recorder, prior to being transcribed verbatim. Transcripts were returned to each participant to enable them to check for accuracy prior to analysis.

Students received a £10 shopping voucher to thank them for their participation in this study in line with INVOLVE guidance [11].

### 2.4 Reflexivity

Interviews were conducted by a junior researcher with no prior research experience. The junior researcher was provided with bespoke training in qualitative interviewing and analysis by the supervising researcher (H.R.), who had significant experience in research and a PhD involving qualitative methodologies. Practice interviews were conducted with feedback to increase the junior researcher's confidence in their interviewing skills.

Both members of the research team were female and had a vested interest in the topic, as their roles involved provision of clinical care which included dietary advice delivery (as a paediatric dentist and foundation therapist, respectively). Having previously experienced learning to provide dietary advice, the researchers also had some insight into the challenges involved with developing these skills.

The interviewer had no involvement with student teaching and hence was not familiar with any of the participants. They were introduced to the student participants as a researcher and wore non‐clinical clothing to avoid introducing any power influence.

### 2.5 Analysis

Data were organised using NVivo software (QSR International PSY Ltd) to support and streamline analysis. Two researchers coded the data independently prior to collaborating to determine sub‐themes and overarching themes [12].

## 3 Results

A total of 11 interviews were carried out with students from Stage 4 and Stage 5 of the BDS course. Of these, five interviews were undertaken with Stage 4 students and six with Stage 5 students. At this stage, no new ideas or concepts were being derived from the interview transcripts, and hence data saturation was considered to have been met. No participants withdrew during the course of this study. No non‐participants were present during any of the interviews, and no repeat interviews were undertaken.

Three overarching themes were identified, which related to managing the child–parent dyad, navigating a patient's daily family life and the learning experience. The coding tree shown in Table 1 outlines the codes, subthemes and themes.

**TABLE 1:** Coding tree outlining the codes, subthemes and themes identified.

Codes	Subthemes	Themes
Anxiety Managing older children Managing younger children Parent engagement Communicating with parents Clinical partners Privacy Levels of comprehension Parents response to advice Busy clinics Walking on eggshells When to involve children and how	Tailoring advice for both Involving the child Challenges from parents Distractions from child	Managing the child–parent dyad
Adherence to diet diary Working with the family Cultural differences Modern family life/structure	Financial viability Cultural competence Complexities of daily life	Navigating daily family life
Availability and accessibility of resources Order of teaching Desire for reassurance pre and post advice delivery Impact of pandemic Confidence Open bay clinics Suggestions for resources Increasing use of technology Reflective learning	Lack of cohesiveness Impact of Covid‐19 Reflective learning Modernisation of resources	Learning experience

### 3.1 Managing the Child–Parent Dyad

Students described difficulties tailoring advice to accommodate both the parent and child's level of comprehension, without the need for repetition:

But, pitching it to both levels without just doing one and then the other, does feel a bit difficult. Participant 3, Stage 5 BDS

Students also struggled to determine when and how to involve a child in diet advice delivery.

I'd say just less involvement of the kid. The kind of the younger they are… they don't have as good development of their communication. Participant 8, Stage 5 BDS

Students felt conscious of their inexperience and found it challenging to deal with negative reactions from parents receiving dietary advice. They also described concern about how parents might perceive their provision of dietary advice, in light of their relative age and lack of seniority.

The mix of not having the confidence, or much experience myself, and the parent being defensive was like a quite a difficult duo…anything I suggested was like shut down… Participant 4, Stage 5 BDS

… it's hard because you're younger giving advice to an older adult…I don't want to sound… that I'm patronising. Participant 6, Stage 5 BDS

Students also highlighted the influence of a child's behaviour on parents' concentration during dietary advice delivery.

… it can be quite distracting for the parent when the child's kind of like doing laps of the dental chair, and getting very agitated at the end, and that's when you're giving diet advice. Participant 4, Stage 5 BDS

### 3.2 Navigating Daily Family Life

Students expressed concerns regarding the financial viability of families actioning the dietary advice that they were delivering and recognised that this may not be feasible for some.

I mean, even just things like, affording food, I get the impression like, some families struggle to buy really healthy fruit and veg and stuff. Whereas I've had patients that, that sort of thing isn't an issue and, they're just somewhat more able to do stuff I guess. Participant 5, Stage 5 BDS

I had recommended some snacks and things and a Mum said that, “Well these just aren't the types of things we have in the cupboard”… And I remember not really knowing what to talk about in terms of the money side of a diet. Participant 3, Stage 5 BDS

Students also highlighted challenges in delivering dietary advice to children and parents from cultural backgrounds that were different to their own, and noted a lack of familiarity with some of the food and drink that these families reported consuming.

I did have an Arabic family who, I thought it was interesting, but it was more difficult, because their diet was not what I was used to. Participant 4, Stage 5 BDS

Students found it difficult to navigate the complexities that different families experience in day‐to‐day life when delivering dietary advice, particularly when the parents had separated, or when grandparents took an active role in childcare.

…also she was separated from the dad and she's like, “Oh, I've told the dad, but, I don't know what happens when she's there. So like, I'm doing my thing, and he's doing his…” Participant 6, Stage 5 BDS

…and “so and so goes to grandmother's on a Tuesday Wednesday Thursday” kind of thing. So that makes it a bit more difficult… Participant 1, Stage 4 BDS

### 3.3 Learning Experience

Students described being reflective in their learning and adapting their delivery of dietary advice as they gained experience.

Some parents will then say, “well what about this?” and then you'll think, “next time I'll add that”. Because you forget. Participant 1 Stage 4 BDS

Students felt there was a lack of cohesiveness in their theoretical and practical learning relating to dietary advice delivery, and recognised the impact of the Covid‐19 pandemic on their experience.

…we got all the teaching and things, then we had a year off or whatever, before we actually used any of it. Participant 8, Stage 5 BDS

Students expressed an awareness of the resources available on the clinic to support and reinforce their delivery of dietary advice and gave insightful suggestions for how these could be modernised and adapted for different age groups.

…the little orange booklets that we give, I feel like they are a bit dated…this generation don't really know Garfield. Participant 5, Stage 5 BDS

if you're talking like teens, even the Garfield like little take home message, I don't think is massively useful. I think you're more going to engage them with the apps, and like leaflets and things. Participant 11, Stage 4 BDS

## 4 Discussion

This novel study explored undergraduate student perceptions of diet advice delivery, revealing the challenges that they experienced and identifying opportunities for their learning to be improved. Through addressing these challenges and building upon these opportunities, there is the potential to improve the student experience of dietary advice delivery, which could encourage them to continue to deliver it upon graduating.

Managing the child–parent dyad was a key challenge identified by participants in this study. Interestingly, the wider literature demonstrates that children have been largely excluded from conversations regarding their health until relatively recently [13, 14]. With the shift towards provision of child‐centred care, it is now increasingly acknowledged that the child's role within their healthcare consultation is key [14]. Furthermore, direct communication between healthcare providers and children has been shown to improve satisfaction with care, adherence to advice and treatment, and ultimately better health outcomes [15, 16]. In oral health research also, parents expressed how involvement of their child in conversations with dental professionals helped to increase their child's awareness of how to care for their own oral health [17]. Nonetheless, it is acknowledged that the practicalities of how and when to involve children in these conversations can be challenging. Research from the medical field concurs with findings from the present study and provides a range of reasons why doctors were less likely to involve children in consultations, including a chaotic environment, lack of time and a lack of skills in communicating with children [13, 18]. The latter is a potential area that could be taught more explicitly within our teaching programmes, alongside managing challenges from parents.

The present study also demonstrated students' concerns regarding the feasibility of families implementing the advice they were delivering, particularly regarding the financial implications. This insight is supported by the literature, which shows that households earning less than £15 860 would have to spend 42% of their disposable income (after housing costs), to meet the national dietary recommendations outlined in ‘The Eatwell guide’ [19, 20]. An adaptation to our teaching to incorporate more affordable healthy snack suggestions for poorer families would be beneficial.

The present study also reported students' perceived lack of cultural competence, which has been defined as “a set of congruent behaviours, attitudes and policies that come together amongst professionals and that enable them to work effectively in cross‐cultural situations” [21]. There is a growing emphasis within dental schools to develop students' ability and skills in providing care for patients and families from diverse cultures [22]. Much of this could be achieved through the decolonisation of dental curricula [23, 24, 25]. It is reported that dietary advice will be less successful if it is not appropriately tailored to the culture of a patient, hence there is a clear need for future efforts to be targeted at this aspect of training in particular [26].

The COVID‐19 pandemic posed significant challenges for dental schools in enabling students to gain sufficient clinical experience to progress with their studies [7]. As such, it is perhaps unsurprising that students highlighted the impact of this on their learning experience in delivering dietary advice. Students also identified the need for dietary advice delivery to be modernised. One proposed change is a move from basing advice upon information collected using a paper 3‐day diet diary to instead using a tablet‐based application to capture a 24‐h diet history. This acknowledges that paper format diet diaries and resources have been found to be an outdated model for delivering diet advice and also recognises the difficulties that parents have reported in completing the diaries alongside busy family life [27, 28].

A key limitation of this study is that no undergraduate dental therapy students were included. The research team initially intended to recruit participants from the undergraduate dental therapy course that runs alongside the dentistry course at Newcastle University. Despite the research team's efforts, no undergraduate dental therapy students expressed an interest in participating. The experiences and perceptions of students training to be dental therapists may differ from those of students on the BDS course, and hence it cannot be assumed that the findings of this study are applicable to therapy students. A further limitation of this study relates to the self‐selection of participants who responded to the invitation email. There is the potential that the experiences of these participants do not reflect those of the students who did not respond to the invitation.

This study has identified a need for advancement in both teaching and research relating to dietary advice delivery. This should focus on supporting students to develop soft skills, using role play and scenarios to prepare them for managing the child–parent dyad and challenging situations. Furthermore, a ‘students as partners’ approach is being employed to co‐create resources to help better prepare students for delivering advice to families from different cultural backgrounds and to families with financial difficulties [29]. Consideration should also be given to the addition of a specific course on cultural competence and interprofessional education on communication strategies.

## 5 Conclusion

Students experience challenges when delivering dietary advice to children and families, particularly regarding engaging parents/carers, determining when and how to involve children of different ages, and giving advice that is sensitive to the complexities of family life, financial difficulties and different cultures. Students undertake reflective, experiential learning when delivering dietary advice, though the pandemic has impacted this. Students have innovative ideas for improving their delivery of dietary advice and should be actively involved in developing future teaching resources.

## Ethics Statement

Ethical approval for this research was granted by Newcastle University Research Ethics Committee. Research was conducted in accordance with the Declaration of Helsinki.

## Consent

Informed consent was obtained from all participants.

## Conflicts of Interest

The author declares no conflicts of interest.

## Supporting information


Data S1.

## Data Availability

Anonymised data are available upon reasonable request to the author.
