# Supplementary material for: Food for Thought: Exploring Dental Students' Perceptions of Delivering Dietary Advice to Children and Families
Source: Eur J Dent Educ. 2025 May 13;30(2):303–8. doi: 10.1111/eje.13118 (PMC13090424; doi:10.1111/eje.13118)
Supplement: Supplementary file 1 — Data S1. [file EJE-30-303-s001.docx]

**Supplementary Material**

**Topic Guide**

**Introduction**

- Confirm with the participant that they have had the opportunity to read the participant information sheet
- Explain to the participant the nature of the study and what it entails: Semi-structured interview with 9 questions to explore their perceptions of delivering diet advice, the effect of the pandemic and interventions they feel that could support their delivery of diet advice
- Encourage participant to be open and honest this interview has no detriment to their clinical or academic progress, but they do not need to discuss something if they don’t want to
- Participants may not compete all 9 questions if multiple questions are answered in one response
- Inform the participant that recording will be via a digital voice recorder that will start only at the point of the first interview question and that interviews will be later transcribed
- Reinform participants of confidentiality and right to withdraw at any stage
- Inform participant of contingency plan to support them if required
- Invite questions from the participant
- Confirm the participant is happy to proceed and sign consent form

**Interview Questions:**

| **Interview Questions** | **Prompts** |
| --- | --- |
| 1. **Can you talk me through your first experience of delivering dietary advice on a paediatric clinic?** | **What was the situation?**  **Who was involved?**  **Where did you source your advice from?**  **How did you feel during and afterwards?** |
| 1. **Can you talk me through how you would usually provide diet advice on clinic to a parent/guardian and patient?** | **Do you think this approach works well?** |
| 1. **How did the parent/guardian respond to the advice you gave?** | **How did this make you feel?** |
| 1. **Did you believe the advice you provided was taken on board?** | **What made you think this?** |
| 1. **Have you provided diet advice on more than one occasion?** | **Is there anything you have done/would do differently when giving dietary advice since/in the future and why?** |
| 1. **How do you feel when you see you have a child who you need to provide diet advice to on clinic?** | **Why do you feel like this?** |
| 1. **What do you think about the clinical environment when delivering dietary advice?** | **Tell me more about why you think this?** |
| 1. **Has there been any impact of the pandemic on your learning regarding dietary advice delivery?** | **Can you explain this in more detail?** |
| 1. **What are your thoughts on using props and visual aids to deliver dietary advice?** | **What aids do you think would change the way you give advice?**  **Do you think this would impact your confidence with delivering advice?** |

**Closing**

- Is there anything you would like to add?

**Follow up**

- Thank the participants for taking part, arrange an appropriate time and date to allow the participant to collect their £10 shopping voucher.
- Reassure participants about confidentiality.
- Re inform participants of the plan to present and publish the results and that this may inform further research. A summary of the analysed data will be shared with the participants in due course.
